# CHEK2 c.1100delC mutation is associated with an increased risk for male breast cancer in Finnish patient population

**DOI:** 10.1186/s12885-017-3631-8

**Published:** 2017-09-05

**Authors:** Sanna Hallamies, Liisa M. Pelttari, Paula Poikonen-Saksela, Antti Jekunen, Arja Jukkola-Vuorinen, Päivi Auvinen, Carl Blomqvist, Kristiina Aittomäki, Johanna Mattson, Heli Nevanlinna

**Affiliations:** 1 Department of Oncology, University of Helsinki and Helsinki University Hospital, Helsinki, Finland; 2 Department of Obstetrics and Gynecology, University of Helsinki and Helsinki University Hospital, Helsinki, Finland; 3 Vaasa Central Hospital, Oncologic Clinic, Turku University, Vaasa, Finland; 4 Department of Oncology and Radiotherapy, Medical Research Center Oulu, Oulu University Hospital and University of Oulu, Oulu, Finland; 5 Department of Oncology, Kuopio University Hospital and Cancer Center, Institute of Clinical Medicine, University of Eastern Finland, Kuopio, Finland; 6 Department of Clinical Genetics, University of Helsinki and Helsinki University Hospital, Helsinki, Finland

**Keywords:** Male breast cancer, CHEK2 c.1100delC

## Abstract

**Background:**

Several susceptibility genes have been established for female breast cancer, of which mutations in *BRCA1* and especially in *BRCA2* are also known risk factors for male breast cancer (MBC). The role of other breast cancer genes in MBC is less well understood.

**Methods:**

In this study, we have genotyped 68 MBC patients for the known breast or ovarian cancer associated mutations in the Finnish population in *CHEK2, PALB2*, *RAD51C*, *RAD51D,* and *FANCM* genes.

**Results:**

*CHEK2* c.1100delC mutation was found in 4 patients (5.9%), which is significantly more frequent than in the control population (OR: 4.47, 95% CI 1.51–13.18, *p* = 0.019). Four *CHEK2* I157T variants were also detected, but the frequency did not significantly differ from population controls (*p* = 0.781). No *RAD51C*, *RAD51D*, *PALB2*, or *FANCM* mutations were found.

**Conclusions:**

These data suggest that the *CHEK2* c.1100delC mutation is associated with an increased risk for MBC in the Finnish population.

**Electronic supplementary material:**

The online version of this article (10.1186/s12885-017-3631-8) contains supplementary material, which is available to authorized users.

## Background

Male breast cancer (MBC) is a rare disease comprising less than 1% of all cancer in men and less than 1% of all breast cancers, but the incidence is increasing [1]. Associated genetic risk factors for MBC are poorly understood. Mutations in *BRCA1* and especially in *BRCA2* are known risk factors, the prevalence varying in different MBC populations [1, 2]. According to a study in 2004, 7.8% of Finnish MBC patients carried a *BRCA2* mutation [3]. In Finland, strong founder effects and enrichment of deleterious alleles are seen [4] and major founder mutations in *BRCA1* and *BRCA2* [5] as well as in other breast and ovarian cancer susceptibility genes account for the vast majority of the identified mutations.

Mutations in cell-cycle checkpoint kinase 2 (*CHEK2*) are associated with an elevated risk for breast cancer [6, 7]. Approximately 4-fold elevated frequency of a protein truncating mutation, c.1100delC in exon 10, was found in Finnish breast cancer patients with a positive family history (5.5%) compared to population controls (1.4%) and a 6-fold in patients with bilateral breast cancer compared to patients with unilateral disease [8]. Among unselected female breast cancer patients, the mutation was observed at a 2.0% frequency. In a previous study of Finnish MBC patients, the frequency of the c.1100delC mutation was similar to that seen in population controls [9] while in a Dutch and an American study, the frequency was significantly elevated [10, 11]. Another variant, the missense I157T (c.470T>C) in the FHA domain of *CHEK2*, is associated with a milder elevation in the risk and was observed in 7.4% of unselected female breast cancer patients, in 5.5% of familial patients, and in 5.3% of population controls [12]. In a recent study [13], the incidence of germline mutations in genes mediating DNA-repair processes among men with metastatic prostate cancer was 11.8%, which was significantly higher than the incidence among men with localized cancer. The results indicated that a *CHEK2* (*p* < 0.001) or a *BRCA2* (*p* < 0.001) mutation may be associated with an elevated risk for a more aggressive type of prostate cancer.

Fanconi anemia is a genetically heterogeneous recessive disorder, which results from biallelic mutations in FA genes, a family of more than 20 genes involved in DNA repair [14]. An increased risk of breast or ovarian cancer is associated with monoallelic mutations in a subset of these genes (*BRCA1*, *BRCA2, BRIP1, PALB2, RAD51*C) [15–20]. In the Finnish population, founder mutations in *BRCA2, PALB2*, and *RAD51C* genes have been identified [5, 18, 21]. *PALB2* c.1592delT mutation has been observed at 0.7–0.9% frequency among Finnish unselected breast cancer patients and at 2.0–2.7% frequency among familial patients and is associated with an aggressive breast tumour phenotype [21, 22]. *RAD51C* mutations c.93delG and c.837+1G>A were observed at a combined frequency of 1.0% among unselected ovarian cancer patients and at 2.1% frequency among breast and ovarian cancer families and associated with an increased risk of ovarian cancer [18]. Neither of the mutations was observed in unselected breast cancer patients. Mutations in *RAD51D* are associated with an increased risk of ovarian cancer [23] and a Finnish founder mutation c.576+1G>A in the gene was significantly more frequent among breast cancer patients with a family history of breast and ovarian cancer (2.9%) than among population controls (0.1%) [24]. Among Finnish unselected ovarian cancer patients, the frequency of the c.576+1G>A mutation was 0.6% whereas the frequency among unselected breast cancer patients (0.1%) was same as in controls [24]. Finally, a *FANCM* nonsense mutation c.5101C>T (p.Q1701X) has been identified as a susceptibility allele for triple-negative breast cancer in the Finnish population and was observed in 2.8% of unselected breast cancer patients and in 3.1% of breast cancer families [25].

Here we have genotyped the *CHEK2* mutations I157T and c.1100delC, the *FANCM* mutation p.Q1701X, the *PALB2* mutation c.1592delT, the *RAD51C* mutations c.837+1G>A and c.93delG, and the *RAD51D* mutation c.576+1G>A in 68 male breast cancer patients. These mutations explain the vast majority of all mutations observed in these genes in the Finnish population, with only a few other, very rare or unique mutations identified.

## Methods

### Patients

We determined the frequency of these mutations in 68 male breast cancer patients. An unselected series of 59 male breast cancer cases was collected at the Helsinki University Hospital Department of Oncology. Altogether 40 patients, diagnosed with breast cancer in 1997–2007 in Helsinki (*n* = 26), Kuopio (*n* = 2), Oulu (*n* = 6) and Vaasa (*n* = 6), were retrospectively collected in 2009–2012. The series included 32% of all male breast cancer cases diagnosed between 1997 and 2007 and 65% of those patients who were alive during the collection period. In addition, 19 patients diagnosed between 2008 and 2013 in Helsinki were collected in 2011–2014 (including 90% of all male breast cancer cases diagnosed in 2008–2013 in Helsinki). One male breast cancer case was identified as part of an unselected series of breast cancer patients collected at the Helsinki University Hospital Department of Surgery in 2001–2004 [26] and eight *BRCA1/2* negative male breast cancer cases diagnosed between 1999 and 2014 were collected at the Helsinki University Hospital Department of Clinical Genetics in 2002–2014. A written, informed consent and a blood sample were obtained from all subjects.

Clinical data including risk factors, patient and primary tumour characteristics were obtained by chart review. Obesity was estimated by calculating body mass index (BMI, > 30 obese) from height and weight recorded in patient charts.

### Genotyping

We genotyped the *RAD51D* c.576+1G>A, *RAD51C* c.93delG, and *RAD51C* c.837+1G>A mutations with Taqman real-time PCR as described elsewhere [18, 24]. The *PALB2* c.1592delT, the *FANCM* c.5101C > T, and the *CHEK2* mutations were genotyped with Sanger sequencing using primer pairs described in Additional file 1 for PCR and ABI BigDyeTerminator 3.1 Cycle Sequencing Kit (Life Technologies) for the sequencing reactions. The capillary sequencing was performed at the Institute for Molecular Medicine Finland (FIMM), University of Helsinki.

For the analysis, we used population control frequencies in the Finnish population defined in previous studies in up to 2102 healthy female population controls from Helsinki (*n* = 1287) and Tampere (*n* = 815) area from the Finnish Red Cross Blood Transfusion Service for the *CHEK2* [8, 12], *RAD51D* [24], *FANCM* [25], and *RAD51C* mutations [18], and 1079 healthy population controls from the Helsinki region for the *PALB2* mutation [22].

The statistical analysis was done using the SPSS 22 for MAC. *P* values for comparisons of male breast cancer patients and population controls were calculated using Fisher’s exact test. All *P* values are two sided.

## Results

The median age at diagnosis of breast cancer was 64 years (range 24–90).

The occurrence of risk factors in the studied patients is presented in Additional file 2. Forty-one patients had been tested for *BRCA* mutations, and of these, 2 (4.9%) were carriers of *BRCA1* mutations and 3 (7.3%) carried a *BRCA2* mutation.

The tumour characteristics are described in Table 1. Nine percent of patients had a T4 tumour. One patient had a lobular carcinoma, one an adenocystic carcinoma, and one a ductal carcinoma in situ whereas all other cancers were ductal carcinomas. Most tumours were hormone receptor positive: 91% were estrogen receptor (ER) positive and 81% progesterone receptor (PR) positive.

**Table 1:** Tumour characteristics

Tumour characteristics		No. (of 68)	Proportion
T-status	T1	36	53%
	T2	20	29%
	T3	0	0%
	T4	6	9%
	Unknown	6	9%
Lymph node status	Positive	22	32%
	Negative	34	50%
	Unknown	12	18%
Histologic tumour type	Ductal	65	96%
	Lobular	1	1%
	Other	2	3%
ER status	Positive	62	91%
	Negative	2	3%
	Unknown	4	6%
PR status	Positive	55	81%
	Negative	6	9%
	Unknown	7	10%
Grade	1	6	9%
	2	30	44%
	3	24	35%
	Unknown	8	12%
Her2	Positive	8	12%
	Negative	42	62%
	Unknown	18	26%

The frequencies of mutations detected in this study and in the control populations are presented in Table 2. The *CHEK2* c.1100delC mutation was found in 4 patients (5.9%) (odds ratio (OR): 4.47, 95% confidence interval (CI) 1.51–13.18, *p* = 0.021 compared to population controls). Median age of the *CHEK2* c.1100delC carriers was 56 years and half of the patients were relatively young at the time of diagnosis, less than 50 years old (Table 3). All the carriers were *BRCA1/2* negative and one patient had a family history of breast cancer. All carcinomas were ductal and estrogen receptor positive. The I157T variant was also detected in 4 patients (*p* = 0.781). No *RAD51C*, *FANCM, PALB2*, or *RAD51D* mutations were found.

**Table 2:** The frequencies of mutations detected in study and control populations

Mutation	This study	Freq^a^	Controls^b^	Freq^c^	*p*-value	OR	95% CI	Ref
*CHEK2* c.1100delC	4 of 68	0.059	26 of 1885	0.014	0.019	4.47	1.51–13.18	8
*CHEK2* I157T	4 of 68	0.059	100 of 1885	0.053	0.781	1.12	0.40–3.13	12
*PALB2* c.1592delT	0 of 68	0	2 of 1079	0.002	1	n.a.	n.a.	22
*FANCM* p.Q1701X	0 of 68	0	38 of 2080	0.018	0.632	n.a.	n.a.	25
*RAD51C* c.93delG	0 of 68	0	2 of 2086	0.001	1	n.a.	n.a.	18
*RAD51C* c.837+1G>A	0 of 68	0	0 of 2086	0	1	n.a.	n.a.	18
*RAD51D* c.576+1G>A	0 of 68	0	1 of 2102	0	1	n.a.	n.a.	24

^a^mutation frequency in this study

^b^see cited reference for control populations

^c^mutation frequency in controls

**Table 3:** Clinical and pathological characteristics of the *CHEK2* c.1100delC carriers

Age range at diagnosis	T	N	M	Histology	ER	PR	Grade	Her2	Family history of breast cancer	Family history of prostate cancer	Family history of ovarian cancer	*BRCA1/2* mutation
65–70	1	1	0	Ductal	Positive	Positive	3	n.a.	n.a.	n.a.	n.a.	Negative
45–50	1	0	0	Ductal	Positive	Negative	2	n.a.	Negative	Negative	Negative	Negative
40–45	1	1	0	Ductal	Positive	Negative	3	Negative	Positive	n.a.	n.a.	Negative
75–80	1	0	0	Ductal	Positive	Positive	2	Positive	Negative	n.a.	n.a.	Negative

## Discussion

The *CHEK2* c.1100delC mutation associated with an increased risk for MBC in the Finnish population of the present study. None of the patients with *CHEK2* c.1100delC mutation had *BRCA1/2* mutation. Mutations in *CHEK2* are known risk factors for female breast cancer [7]. The 5.9% mutation frequency detected among the male breast cancer patients is higher than among the unselected female patients and comparable to that among female familial cases (5.5%) [8]. Similar to female breast cancer [27], the male *CHEK2* c.1100delC carrier tumours were of ductal histology, ER positive, and poorly differentiated (grade 2–3). The *CHEK2* c.1100delC mutation has been also linked to an increased risk for prostate cancer [28], and possibly indicates an elevated risk for metastatic prostate cancer [13].

The role of the *CHEK2* c.1100delC mutation as a risk factor for MBC has been studied in several papers [6, 9–11, 29–32]. An association between *CHEK2* c.1100delC mutation and MBC risk has been reported in three studies [6, 10, 11]. A wide variation in the population frequency of c.1100delC has been observed, with highest reported frequencies in the Netherlands (1.3–1.6%) and in Finland (1.1–1.4%) [7]. The rarity of MBC and the geographic variation in the frequency of the *CHEK*2 c.1100delC mutation might explain the differences between studies on the possible association between the *CHEK2* c.1100delC mutation and MBC. In a previous Finnish study, the *CHEK2* c.1100delC mutation frequency was not significantly elevated in MBC patients, and did not seem to affect the age on breast cancer onset [9]. Only 10.5% of patients in that study had a family history of female breast or ovarian cancer. In our study, however, the *CHEK2* c.1100delC was significantly more frequent in the studied population compared to population controls, despite the smaller sample size. The median age of the patients with *CHEK2* c.1100delC mutation was also younger than in the whole population studied. Of the 43 patients with known family history, 25% had a positive family history of female breast cancer. The population control frequency to which the previous study compared their results was the same as the one used in this study. Our finding is in line with the Dutch study where the frequency of the c.1100delC mutation was significantly elevated compared to that seen in population controls (OR: 4.1, 95% CI 1.2–14.3, *p* = 0.05) [10], both studies suggesting about four- to five-fold elevated risk for male breast cancer for the mutation carriers. Very recently, a large American multi-gene panel study with 715 MBC patients showed that *CHEK2* c.1100delC was associated with moderately increased risks of MBC (OR: 3.8, 95% CI 1.7–7.8) [11].

Mutations in *RAD51C* and *RAD51D* are rare and have been primarily linked to an increased risk for ovarian cancer [19, 23] rather than breast cancer alone. Previously, no truncating *RAD51C* mutations were identified among 97 Italian MBC patients and the American multi-gene panel study also did not identify any *RAD51C* mutations whereas one *RAD51D* mutation was observed among the 715 MBC patients [11, 33]. *PALB2* mutations among MBC patients are relatively rare (0.8%) in the US population but have been associated with a significantly increased risk of male breast cancer [11]. In this study, we did not detect the *RAD51C, RAD51D, FANCM*, or *PALB2* mutations among male breast cancer patients. However, our sample size was small. Given the relatively low frequency of *RAD51C*, *RAD51D*, *FANCM*, and *PALB2* mutations among unselected female breast cancer patients, the absence of the studied mutations in the MBC series was not unexpected. Larger studies are warranted to better evaluate the contribution of these genes to MBC susceptibility in the Finnish population.

## Conclusions

In conclusion, the *CHEK2* c.1100delC mutation is associated with an increased risk for MBC in the present study in Finnish population. In light of previous data from the Netherlands [10] and USA [11], our study thus suggests that inclusion of *CHEK2* mutation analyses should be considered as a part of genetic testing of MBC patients, at least in populations with prevalent mutations.

## Additional files

Additional file 1: Primer pairs used in the genotyping of the *CHEK2* c.1100delC and I157T, *PALB2* c.1592delT and *FANCM* c.5101C>T mutations. (DOCX 13 kb)

Additional file 2: The occurrence of risk factors for male breast cancer. (DOCX 12 kb)

## Abbreviations

BMI: Body mass index
CI: Confidence interval
ER: Estrogen receptor
MBC: Male breast cancer
OR: Odds ratio
PR: Progesterone receptor
