# Supplementary material for: CHEK2 c.1100delC mutation is associated with an increased risk for male breast cancer in Finnish patient population
Source: BMC Cancer. 2017 Sep 5;17:620. doi: 10.1186/s12885-017-3631-8 (PMC5584025; doi:10.1186/s12885-017-3631-8)
Supplement: Supplementary file 1 — Primer pairs used in the genotyping of the CHEK2 c.1100delC and I157T, PALB2 c.1592delT and FANCM c.5101C>T mutations. (DOCX 13 kb) [file 12885_2017_3631_MOESM1_ESM.docx]

**Additional file 1.** Primer pairs used in the genotyping of the *CHEK2* c.1100delC and I157, *PALB2* c.1592delT and *FANCM* c.5101C>T mutations.

| **Mutation** | **Forward primer** | **Reverse primer** | **T (°C)** |
| --- | --- | --- | --- |
| *CHEK2* c.1100delC | 5'-TTAATTTAAGCAAAATTAAATGTCC-3' | 5'-GGCATGGTGGTGTGCATC-3' | 55 |
| *CHEK2* I157T | 5'-GGCTATTTTCCTACAATTAGC-3' | 5'-CATATTCTGTAAGGACAGGAC-3' | 55 |
| *PALB2* c.1592delT | 5'-CCAAGTTCAAGAACCTCTCAG-3' | 5'-GCAAATAGTAATTGTTAACTTTCATCA-3' | 55 |
| *FANCM* c.5101C>T | 5'-GAGTCTTGCAAAGGCCAATC-3' | 5'-TCAGCGATGTCTGTTTGCTC-3' | 58 |

**T = annealing temperature**
