# Supplementary material for: CHEK2 c.1100delC mutation is associated with an increased risk for male breast cancer in Finnish patient population
Source: BMC Cancer. 2017 Sep 5;17:620. doi: 10.1186/s12885-017-3631-8 (PMC5584025; doi:10.1186/s12885-017-3631-8)
Supplement: Supplementary file 2 — The occurrence of risk factors for male breast cancer. (DOCX 12 kb) [file 12885_2017_3631_MOESM2_ESM.docx]

**Additional file 2:** The occurrence of risk factors for male breast cancer

| **Risk factors** | **No.** | **Proportion of known** |
| --- | --- | --- |
| **Obesity (BMI >31)**  Obese  Normal weight | Known in 32  9  23 | 28%  72% |
| **Alcohol intake**  None  Occasional  High | Known in 27  6  6  15 | 22%  22%  56% |
| **Liver cirrhosis** | 3 | 5% |
| **Family history of breast cancer**  Negative  Positive | Known in 42  25  17 | 60%  40% |
| **Family history of prostate cancer**  Negative  Positive | Known in 35  31  4 | 89%  11% |
| **Family history of ovarian cancer**  Negative  Positive | Known in 36  36  0 | 100%  0% |
| **BRCA mutation**  BRCA1  BRCA2  No BRCA mutation | **No. (of 41 tested)**  2  3  36 | 4.9%  7.3%  87.8% |
| **Klinefelter’s syndrome** | 0 | 0% |
